# COVID-19 vaccine eligibility of pregnant and lactating women in Bangladesh: Gap between policy and policy interpretation among policymakers and healthcare workers

**DOI:** 10.1016/j.jvacx.2023.100370

**Published:** 2023-08-09

**Authors:** Rupali J. Limaye, Berhaun Fesshaye, Prachi Singh, Eleonor Zavala, Shirina Akter, Towfida Jahan Siddiqua, Hafizur Rahman, Hasmot Ali, Ruth Karron

**Affiliations:** a Department of International Health, Johns Hopkins University, Bloomberg School of Public Health, Baltimore, MD, USA; b International Vaccine Access Center, Johns Hopkins University, Bloomberg School of Public Health, Baltimore, MD, USA; c Department of Health, Behavior & Society, Johns Hopkins University, Bloomberg School of Public Health, Baltimore, MD, USA; d Department of Epidemiology, Johns Hopkins University Bloomberg School of Public Health, Baltimore, MD, USA; e JiVitA Project, Johns Hopkins University, Rangpur, Bangladesh

**Keywords:** Vaccination, Pregnant women, Bangladesh, Healthcare workers, COVID-19

## Abstract

SARS-CoV-2 infection in pregnancy is associated with a greater risk of maternal and newborn morbidity and maternal death. Bangladesh confirmed its first COVID-19 case in March of 2020, and vaccination rollout started in January of 2021. In Bangladesh, pregnant women are allowed to receive COVID-19 vaccines during pregnancy with qualifications while lactating women are permitted to receive COVID-19 vaccines with no qualifications as of October 2021. There is limited evidence on how vaccine policies are disseminated, interpreted, and implemented from the national level to the community level in Bangladesh. We conducted in-depth interviews from April-August 2022 with policymakers and healthcare workers in Bangladesh to understand how different stakeholders understood and implemented COVID-19 vaccination policies related to pregnant and lactating women. We interviewed policymakers at three levels: national, divisional, and district, and interviewed healthcare workers from one one urban and three rural communities within one division. We found a gap between policies related to COVID-19 vaccination for pregnant and lactating women and policy interpretation among policymakers and healthcare workers. Policymakers and healthcare workers’ perceptions differed related to policy dissemination, attitudes toward policies related to pregnant and lactating women, and eligibility of pregnant and lactating women. Our findings indicate the need for effective dissemination of and understanding of policies. Within the context of vaccine uptake and vaccine acceptance, policymakers play a critical role as they are charged with developing and disseminating policy related to vaccine eligibility. Healthcare workers rely on timely and accurate communication related to vaccine eligibility, including populations, timing, and locations. Efforts are needed to narrow the policy and policy implementation gap as doing so is crucial to controlling vaccine preventable disease.

## Introduction

The first case of COVID-19 in Bangladesh was confirmed on March 8, 2020, and since then, over two million cases and almost 30,000 deaths have been documented [22]. To mitigate the initial wave of the pandemic in the absence of vaccines, the government of Bangladesh imposed lockdowns and opened specialized COVID-19 treatments units across the country [13].

As the first COVID-19 vaccines became available, Bangladesh signed up to receive supply support from the COVAX initiative as well as vaccine producers around the world [13]. A variety of different vaccines have been approved for use in Bangladesh with their vaccine platforms including protein subunit, RNA, non-replicating viral vector, and inactivated (COVID-19 Vaccine Tracker, 2022). COVID-19 vaccination began in Bangladesh on January 27, 2021, with prioritization of healthcare workers, frontline workers, and individuals above the age of 55 [22], [18]. Eligibility was expanded to adults over the age of 18 in October 2021 (Dhaka [5]). Over 353 million doses have been administered to date with 79% of the adult population being fully vaccinated, excluding booster doses [9], [22].

As COVID-19 vaccine clinical trials did not include pregnant and lactating women, data on the safety and effectiveness of COVID-19 vaccines in these populations were not available at the outset of the global vaccine campaign. However, evidence showed pregnant women infected with SARS-CoV-2 were at higher risk of severe disease compared to non-pregnant women[1], and at higher risk of adverse birth outcomes, including preterm birth, compared to non-infected pregnant women[10]. As vaccine uptake increased globally, observational data on the use of COVID-19 vaccines in pregnant women indicated no increased risk of adverse events with vaccination [14], and provided protection for both mother and infant against severe illness and hospitalization [11], [7].

At the outset of the COVID-19 vaccine campaign in Bangladesh, pregnant and lactating women were ineligible to receive a vaccine, due to the lack of vaccine safety data in this population. In October 2021, the Bangladesh Directorate General of Health Services issued a memorandum permitting the administration of COVID-19 vaccines in pregnant women, after receiving counseling from a physician and signing a consent form. The letter cited the recommendation from the National Technical Advisory Committee (NTAC), the risks of COVID-19 during pregnancy, and the benefits of vaccination as indications for the change in policy. Lactating women could begin to receive vaccines without additional qualifications.

Vaccine policy is an important cornerstone for access to vaccines, including eligibility. Vaccine policy provides a framework for recommendations and delivery; however, understanding how various stakeholders understand and interpret policy is critical for uptake. We were interested in better understanding the policy related to pregnant and lactating women, and as such, we focused this analysis on perception of the policy and interpretation, comparing perceptions between policymakers and healthcare workers.

## Materials and methods

**Fig. 1:**
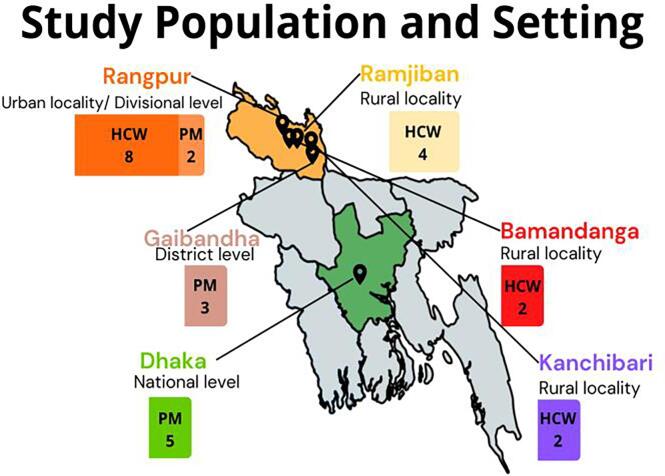
Map of sampled populations and locations across Bangladesh (n = 26 interviews; 16 healthcare workers and 10 policymakers).

This cross-sectional qualitative study interviewed policymakers and healthcare workers in Bangladesh. Identification of relevant policymakers was conducted by our local collaborating organization, JiVitA, in consultation with colleagues from Jhpiego Bangladesh. JiVitA is the Maternal and Child Health and Nutrition project site of the Johns Hopkins Bloomberg School of Public Health located in Bangladesh. Policymakers were recruited at the national, divisional, and district levels from Dhaka, Rangpur division, and Gaibandha district. Healthcare worker participants were recruited at health facilities from four different communities in Rangpur Division in northern Bangladesh: Rangpur city (urban), Bamandanga (rural), and Ramjiban (rural). See Fig. 1 for locations of study participants. Data were collected between April-August 2022. Interview instruments included questions related to COVID-19 vaccine policies for pregnant and lactating women. Data collectors with qualitative research experience participated in a two-day training including human subjects research ethics, qualitative research techniques, and field testing. Oral consent was obtained from those who expressed interest in joining the study. Interviews were conducted in Bangla in semi-private settings at the health facility or at the office of the policymaker. All interviews were audio recorded, then later transcribed, and translated to English by members of the study team and external translators fluent in both languages. All data, including audio recordings, were stored on encrypted servers, and only members of the study team had access to the data.

A team of 7 used a grounded theory approach to analyze the data. Data were managed using Atlas.ti. The code list was developed, refined, and finalized over three rounds of open coding. Following agreement of a code list, the team coded the transcripts, holding discussions on emerging themes after coding ∼ 25% and 50% of the transcripts. Two members of the team conducted inter-rater reliability with ∼ 10% of the transcripts that neither of them had coded (3 transcripts). Reliability was 93%. The team then identified themes and sub-themes. This study received ethical approval from (*blinded for review*) and (*blinded for review*).

## Results

We interviewed 16 healthcare workers (8 that served rural communities and 8 that served urban communities), and 10 policymakers that were from three different levels of the health system: national, divisional, and district, for a total of 26 interviews.

**Fig. 2:**
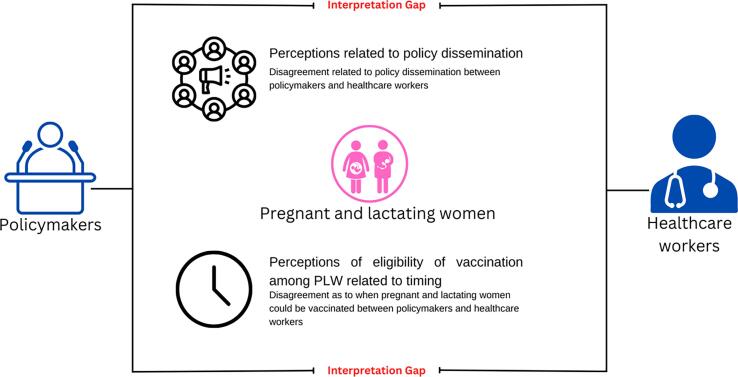
Gaps in interpretation related to policy dissemination and COVID-19 vaccine timing for pregnant and lactating women.

Three key themes emerged, and there was an interpretation gap within two of the three themes. Themes included perceptions related to policy dissemination, attitudes toward policies related to pregnant and lactating women eligibility, and perceptions of eligibility of vaccination among pregnant and lactating women related to timing (Fig. 2).

## Policy dissemination & attitudes toward policy

There was a stark difference in perceptions related to policy dissemination. While policymakers believed that the policy related to vaccinating pregnant and lactating women was disseminated widely, healthcare workers did not have the same perception. This policymaker asserted: *“Dissemination is always a hard job to do, it is really hard to reach in all in a rural community! But it was broadcasted in every channel (TV), scrolling was going on where the government said that pregnant and lactating women should get the vaccine. Our government issued a notification/communique which said ‘each pregnant and lactating, nursing mother must take the vaccine - vaccination is also for pregnant women.’ We received published guidelines, the notification/communique was distributed in all districts and at the Upazilla level”* (National-level policymaker). Healthcare workers overwhelmingly reported that they received no written guidelines and stated that they only received instructions related to vaccinating pregnant and lactating women verbally: “I *have the question in my mind, that why did they say first that you cannot give the vaccine to pregnant women. Since we did not get training, or get these instructions in writing, they only locally said one day that now we can give it to pregnant women****.*** *So, if there was more training, then we would understand even better. More training would be good. Instead of just being given verbal instructions*” (Healthcare worker, rural Bamandanga). This healthcare worker alluded to the fact that the dissemination of the policy to lower levels of the health care system was slow: “*Maybe in the Upazila health complex when they are instructed by their authority to change the policy… then they instruct us…These all are under experiments, experiments are ongoing…It continues. Research continues in the lab…then it is released…gradually that comes to the lower level of the health department…We get the instructions then*” (Healthcare worker, rural Kanchibari).

Policymakers believed that healthcare workers and pregnant women would accept any policy related to the eligibility of pregnant and lactating women: “*Healthcare workers accept it cordially because it is their job. It’s normal to follow upper-level instructions. There is no chance for them to perceive it negatively. Pregnant and lactating women, I think they received it as I receive it. Everyone received it in the same way*” (District-level policymaker). Policymakers also believed that healthcare workers would positively accept the policy: “*Health Care Providers take the policy positively, I believe. Because they also have to work with these mothers. Vaccinated mothers are naturally safer than others (who are not vaccinated). So, they also feel relieved during providing care that the mother is vaccinated. That means the risk of the mother is less as well as the service provider if they come in contact with each other*” (Divisional-level policymaker). Healthcare workers accepted the policy positively. When asked what they thought about the policy related to pregnant and lactating women, this was their response: “*I think it’s a good policy. Because definitely, the scientist finds out some good results after research, that’s how they make the policy. They are giving that vaccine after the research*” (Healthcare worker, rural Bamandanga).

## Eligibility of pregnant and lactating women: Timing

Among policymakers, there were conflicting perceptions regarding when pregnant and/or lactating women could receive the vaccine. For example, this divisional-level policymaker understood that the vaccine was given to these women only within a certain time: *“Pregnant and lactating mothers were not getting the vaccine from the very beginning…There is a limitation of vaccination in first trimester and the last trimester, I exactly cannot recall it. Vaccine is not given in the first trimester as pregnancy is in a vulnerable stage at this period. The vaccination program started last year (2021). Present vaccination policy is that pregnant and lactating mothers can avail or take the vaccine in their eligible period”* (Divisional-level policymaker). While this policymaker understood that specifically related to pregnant women, they could receive the vaccine anytime during their pregnancy: *“There was speculation and imagination about it, ‘should it be given during the first trimester or not? We informed healthcare workers later that the vaccine can be received at any time, in any trimester”* (National-level policymaker).

From the healthcare worker perspective, there was no agreement regarding the time in which a pregnant woman was eligible to receive the vaccine. Some referred to eligibility being the same as when the tetanus toxoid vaccine is given during pregnancy; the government of Bangladesh approved the TT5 dose in 1993 [15]. This healthcare worker explained that eligibility was only between 4 and 8 months of the pregnancy: *“There is a misconception among the people…If we give any vaccine, anytime you can take tetanus toxoid vaccine…I am giving you an example through the tetanus toxoid vaccines…If there is any abortion happened then all the fault came upon us…Yes, That’s why we tell them to take it after four months. There is reliability after four months. We tell them to take four months of gestation period…If the abortion happened for another reason, then the fault also came upon us. So, we give from four months to eight months. We do not give before four months or after 8 months”* (Healthcare worker, rural Kanchibari). This healthcare worker explained that eligibility was only between 4 and 7 months of the pregnancy: *“Unless it has been 4 months, we don’t give the vaccination. And then right before delivery we don’t give it. If it has been 7 months, then we don’t give the vaccination…we recommend to the mother to come back after the delivery and then take it. Just like the tetanus toxoid vaccination, we do the same with Corona vaccine. That if we give it before 3 months, and they haven’t conceived, then they get their menstruation, they might think that they had an abortion because of the vaccination. And then again if we give the vaccine after 8 months of pregnancy, in that case, say the baby is born with some kind of problem, or, god forbid, if the baby is stillborn, in these cases, they might think that the vaccination is the reason that they lost their baby. This is true for tetanus toxoid vaccination, and we apply it for Corona vaccination also”* (Healthcare worker, rural Bamandanga). This healthcare worker believed women were eligible between 4 and 6 months of pregnancy: *It is not given before delivery and during the first trimester. It is given in four to six months. It is given four to six months but not given before delivery (third trimester)* (Healthcare worker, rural Ramjiban). Finally, this healthcare worker was not aware of the restrictions related to vaccine eligibility: “*No, there are no rules…we give to everyone. No, there are no rules, we give it to everyone who comes here*” (Healthcare worker, rural Kanchibari).

Specific to lactating women, healthcare workers had different perceptions regarding timing of vaccine eligibility. This healthcare worker thought the mother was eligible two months after giving birth: “*Since there is provision that the mother can take it after delivery, maybe like 2 months after delivery, once your baby is already out*” (Healthcare worker, rural Bamandanga). This healthcare worker noted that they were not sure if the vaccine could be given if the lactating woman’s baby was less than two months old: “*Vaccine is given to the mothers who have a two- or three-months old baby. I do not know if it is given to mothers who have smaller kids than that*” (Healthcare worker, rural Ramjiban). Finally, this healthcare worker was not aware of any rules related to timing after giving birth: “*We are vaccinating the lactating mother. We are giving them after the baby is born. There are no exact rules in their case (when the vaccine can be given after birth). They can be vaccinated after the delivery anytime*” (Healthcare worker, rural Kanchibari). This healthcare worker also believed that there were no rules related to timing after giving birth: *There is no direction also about the lactating mother. That’s why every mother can take the vaccines”* (Healthcare worker, rural Bamandanga).

This healthcare worker discussed timing eligibility related to pregnant and lactating women within the context of vaccine product: “*So first, we got AstraZeneca. Because of AstraZeneca, you have high fever. Then we got the instruction not to give the vaccine to the pregnant and breastfeeding mothers…since high fever was a side effect of AstraZeneca. Then the people did research and found out that it is okay to give this to pregnant mothers and breastfeeding mothers. But, if you give it to breastfeeding mothers, then maybe the breastfeeding baby might also get the fever. So, when the baby will eat something else, other than drinking mother’s milk, in those cases, we can give the covid vaccine, but for 24 h, the baby cannot be breastfed. First this was the instruction. Then after that, the instruction was 12 h, not 24 h. Then a vaccine came that was called Sinovac or Sinopharm, from China. That vaccine had very little side effect, almost none. So, for that, the instruction was that, we can give it right after delivery, you can give it 2*–*3 months after delivery, and you can give it to pregnant mothers too. Since there are no side effects, you can do that, it will not affect the mother or the baby. This is what our supervisors explained us. This was for Sinovac. But before when we had given AstraZeneca, the instruction was that, since this has side effect, we are better off not giving it to pregnant and breastfeeding mothers. They can take it later on”* (Healthcare worker, rural Bamandanga).

## Discussion

Our results illustrate the gap between policy and policy interpretation and understanding, as well as the importance of policy dissemination and communication. Among policymakers, there was no agreement regarding timing eligibility of pregnant and lactating women for the vaccine. There were even more disagreements among healthcare workers related to timing eligibility. However, there were generally positive attitudes toward the policy among policymakers and healthcare workers. As the COVID-19 pandemic was constantly evolving, the policy environment related to vaccine eligibility was also quite dynamic [25]. Many countries had similar challenges related to a gap between policy and policy interpretation (see Kenya, for example) [24].

In times of crisis, such as a pandemic, effective communication is a crucial fundamental skill required of those in the health care system [6]. From a policymaker perspective, this includes the dissemination of accurate and timely information delivered efficiently and consistently [17]. Policymakers play a critical role in providing clear communication about policies, and this is especially pertinent within a pandemic [4]. It is important for governments to balance precision and clarity when developing vaccine policies as increased precision may lead to greater complexity and lower policy comprehension [16]. Government authorities must disseminate information through multiple channels for all audiences, including healthcare workers [3]. It is also important to note that without adequate vaccine safety data specific to pregnant women, which many policymakers utilize to make recommendations, confusion can occur during the policy formulation phase even before communication and dissemination begin [24]. Therefore, inclusion of pregnant and lactating populations in vaccine clinical trials to ensure appropriate data exists prior to vaccine rollout is critical [24].

Unclear policy guidance posed challenges for both policymakers and healthcare workers in our study. As healthcare workers are consistently cited as a trusted source of information for vaccination [8], [12], their interpretation of a policy is paramount for vaccine acceptance [21]. Not only must healthcare workers be aware of the policy and understand it, but they also must be adequately trained to confidently counsel women, allowing them to make informed decisions regarding vaccination [20], [23]. However, inadequate communication networks and flow of information throughout the healthcare system can hinder healthcare workers’ ability to assist in decision-making and make a strong recommendation for their clients [2].

While lack of clarity related to policy eligibility affects policymaker and healthcare workers perceptions, their misunderstanding can have downstream effects. If there is confusion related to eligibility, this will affect uptake of pregnant and lactating women. Additionally, if pregnant and lactating women feel as though they cannot trust healthcare workers or government authorities broadly, this affects their perception of adherence to other recommendations [19].

A potential solution for addressing the gap in health communication is the use of social media and mobile phone technology, given the wide reach, with many rural communities in LMICs having more than 80% access to mobile phone networks in contrast to the limited access to landline media channels (Milden and Sellen, 2019; Islam et al., 2020). While risks such as misinformation exist, social media can work to improve health information sharing and aid in public health campaigns (Islam et al., 2020). SMS messages through mobile phones providing education and reminders have shown mostly positive effects on care-seeking behaviors, such as immunization (Milden and Sellen, 2019).

This study is not without limitations. This was a cross-sectional qualitative study, and results are not generalizable. However, we still believe that our exploratory results have value in the field of maternal vaccine policy. As the COVID-19 situation was ever-changing, policies were also changing. How policy change was disseminated through different levels of the health care system in the country likely varied greatly. However, this allowed us to examine and identify a gap between policy and policy interpretation among both policymakers and healthcare workers. This study has several strengths. We used a holistic approach to understand vaccine policy focused on pregnant and lactating women, as we interviewed policymakers and healthcare workers. Our study is one of the first to examine COVID-19 vaccine policies related to pregnant and lactating women, and our findings can uniquely inform how future maternal immunization policy is introduced, communicated and implemented among key target audiences.

It is crucial that pregnant and lactating women are provided evidence-based information about their eligibility to receive a COVID-19 vaccine. Policymakers play an essential role given their role in developing and disseminating policy related to vaccine eligibility. Healthcare workers and the health care system broadly rely on clear communication about who can be vaccinated, when they can be vaccinated, and how they can be vaccinated. In addition, healthcare providers that have the trust of their patients and one-on-one conversations with them regularly are uniquely situated to increase vaccine acceptance among these populations. As COVID-19 will likely not be the last pandemic we experience in our lifetimes, disseminating policy including eligibility considerations must be timely, accurate, and clear to be able to reduce the morbidity and mortality of disease. We hope that others use our results to inform the design of communication strategies for policy rollout and policy dissemination related to maternal vaccination.

## Funding

This research was funded by the Bill & Melinda Gates Foundation, grant number INV-016977.

## Declaration of Competing Interest

The authors declare that they have no known competing financial interests or personal relationships that could have appeared to influence the work reported in this paper.

## Data Availability

Data will be made available on request.

## References

[1] Allotey J., Stallings E., Bonet M., Yap M., Chatterjee S., Kew T. (2020). Clinical manifestations, risk factors, and maternal and perinatal outcomes of coronavirus disease 2019 in pregnancy: living systematic review and meta-analysis. BMJ.

[2] Al-Zaman M.S. (2020). Healthcare Crisis in Bangladesh during the COVID-19 Pandemic. Am J Trop Med Hyg.

[3] Berg S.H., O’Hara J.K., Shortt M.T., Thune H., Brønnick K.K., Lungu D.A. (2021). Health authorities’ health risk communication with the public during pandemics: a rapid scoping review. BMC Public Health.

[4] Childress MT, Clark MW. Communicating with Policymakers in a Pandemic. Communicating Science in Times of Crisis: The COVID‐19 Pandemic, 2021:321–37.

[5] Dhaka Tribune*.* (2021). Bangladesh lowers Covid-19 vaccine age limit to 18 years. *Dhaka Tribune*. Accessed February 7, 2023. Available from: https://www.dhakatribune.com/bangladesh/2021/10/20/bangladesh-lowers-covid-19-vaccine-age-limit-to-18-years.

[6] Eldridge C.C., Hampton D., Marfell J. (2020). Communication during crisis. Nurs Manage.

[7] Halasa N.B., Olson S.M., Staat M.A., Newhams M.M., Price A.M., Boom J.A. (2022). Effectiveness of maternal vaccination with mRNA COVID-19 vaccine during pregnancy against COVID-19–associated hospitalization in infants aged< 6 months—17 states, July 2021–January 2022. Morb Mortal Wkly Rep.

[8] Healy C.M., Rench M.A., Montesinos D.P., Ng N., Swaim L.S. (2015). Knowledge and attitudes of pregnant women and their providers towards recommendations for immunization during pregnancy. Vaccine.

[9] Mathieu E, Ritchie H, Rodés-Guirao L, Appel C, Giattino C, Hasell J, Macdonald B, Dattani S, Beltekian D, Ortiz-Ospina E, Roser M. Coronavirus Pandemic (COVID-19). Our World in Data. Accessed February 8, 2023 (2020). Available from: https://ourworldindata.org/covid-vaccinations.

[10] McClymont E., Albert A.Y., Alton G.D., Boucoiran I., Castillo E., Fell D.B. (2022). Association of SARS-CoV-2 infection during pregnancy with maternal and perinatal outcomes. J Am Med Assoc.

[11] Morgan J.A., Biggio J.R., Martin J.K., Mussarat N., Chawla H.K., Puri P. (2022). Maternal outcomes after severe acute respiratory syndrome coronavirus 2 (SARS-CoV-2) infection in vaccinated compared with unvaccinated pregnant patients. Obstet Gynecol.

[12] Nowak S.A., Gidengil C.A., Parker A.M., Matthews L.J. (2021). Association among trust in health care providers, friends, and family, and vaccine hesitancy. Vaccine.

[13] Rahaman P. Bangladesh’s success in dealing with COVID-19. Gavi, The Vaccine Alliance. Accessed February 7, 2023 (2022). Available from: https://www.gavi.org/vaccineswork/bangladeshs-success-dealing-covid-19.

[14] Sadarangani M., Soe P., Shulha H.P., Valiquette L., Vanderkooi O.G., Kellner J.D. (2022). Safety of COVID-19 vaccines in pregnancy: a Canadian National Vaccine Safety (CANVAS) network cohort study. Lancet Infect Dis.

[15] Sarkar P.K., Kumar Sarker N., Doulah S., Bari T.I.A. (2015). Expanded programme on immunization in Bangladesh: a success story. Bangladesh J Child Health.

[16] Schurr E.H., Luisi N., Sanchez T., Lopman B.A., Bradley H., Sullivan P.S. (2022). Association of Guideline Complexity With Individuals’ Ability to Determine Eligibility for COVID-19 Vaccination. JAMA Netw Open.

[17] Simonovich S.D., Spurlark R.S., Badowski D., Krawczyk S., Soco C., Ponder T.N. (2021). Examining effective communication in nursing practice during COVID-19: A large-scale qualitative study. Int Nurs Rev.

[18] United Nations Bangladesh. (2021). UN Bangladesh COVID-19 Quarterly Situation Report- Q4 2020. *United Nations in Bangladesh*. Accessed February 7, 2023. Available from: https://bangladesh.un.org/en/116428-un-bangladesh-covid-19-quarterly-situation-report-q4-2020.

[19] Varghese N.E., Sabat I., Neumann-Böhme S., Schreyögg J., Stargardt T., Torbica A. (2021). Risk communication during COVID-19: A descriptive study on familiarity with, adherence to and trust in the WHO preventive measures. PLoS One.

[20] Wallace A.S., Ryman T.K., Dietz V. (2012). Experiences integrating delivery of maternal and child health services with childhood immunization programs: systematic review update. J Infect Dis.

[21] Wilson R.J., Paterson P., Jarrett C., Larson H.J. (2015). Understanding factors influencing vaccination acceptance during pregnancy globally: a literature review. Vaccine.

[22] World Health Organization (2023). Bangladesh: WHO Coronavirus Disease (COVID-19) Dashboard With Vaccination Data. https://covid19.who.int/region/searo/country/bd.

[23] Yuen C.Y.S., Tarrant M. (2014). Determinants of uptake of influenza vaccination among pregnant women–a systematic review. Vaccine.

[24] Zavala E., Fesshaye B., Lee C., Mutwiwa S., Njagi W., Munyao P. (2022). Lack of clear national policy guidance on COVID-19 vaccines influences behaviors in pregnant and lactating women in Kenya. Hum Vaccin Immunother.

[25] Zavala E., Krubiner C.B., Jaffe E.F., Nicklin A., Gur-Arie R., Wonodi C. (2022). Global disparities in public health guidance for the use of COVID-19 vaccines in pregnancy. BMJ Glob Health.

